# Reliability-Aware Neural Decoding with Adaptive Multi-Source Information Fusion

**DOI:** 10.3390/e28030323

**Published:** 2026-03-13

**Authors:** Pengxi Fu, Zhen Wang, Jianxin Guo, Yushuai Zhang, Feng Wang, Rui Zhu, Zhentao Huang

**Affiliations:** 1 Xi’an Key Laboratory of Intelligent Perception and Autonomous Navigation for Low-Altitude Aircraft, Xijing University, Xi’an 710123, China; ldpc_res@163.com (P.F.); guojx_516@126.com (J.G.); apacheyu@yeah.net (Y.Z.); wangfengisn@163.com (F.W.); zhu_r10@163.com (R.Z.);; 2 School of Computer Science, Northwestern Polytechnical University, Xi’an 710129, China

**Keywords:** neural decoding, multi-source information fusion, reliability-aware gating, heterogeneous message passing, adaptive weighting, deep feature injection, robustness to mismatch

## Abstract

Modern communication systems increasingly leverage multiple information streams—including channel observations, statistical models, and contextual knowledge—to enhance decoding reliability. However, the varying and often unpredictable quality of these sources poses a critical challenge: rigid combination rules fail when source reliability fluctuates, while manual tuning cannot adapt to dynamic operating conditions. This paper presents a neural decoder architecture that automatically learns to assess and fuse heterogeneous information sources based on their instantaneous reliability. Central to our design is a learnable gating module that dynamically weights information streams, demonstrating emergent Bayesian-like behavior—increasing reliance on statistical models under high uncertainty while transitioning to observation-dominated processing as signal confidence improves. To combat the progressive dilution of auxiliary information in deep architectures, we propose a continuous injection strategy that refreshes auxiliary features at each processing layer through dedicated encoding pathways. The underlying message-passing network adopts a heterogeneous bipartite structure with direction-dependent edge parameterization, respecting the asymmetric computational roles inherent in iterative decoding algorithms. Comprehensive experiments validate that the proposed approach not only improves nominal performance but critically maintains robustness when auxiliary information quality degrades or becomes mismatched with actual conditions.

## 1. Introduction

In modern communication architectures, uncompressed textual data frequently passes directly to the physical layer [1], where it occupies significant capacity despite its high inherent compressibility. The absence of native compression support at this layer means that transmitting such raw information inefficiently consumes both bandwidth and electrical power, ultimately degrading system-wide throughput.

As wireless networks evolve toward the 6G era, the demand for significantly higher spectral efficiency continues to intensify [2], with emerging upper mid-band spectrum allocations presenting both new opportunities and challenges [3]. Within this context, exploiting all available information sources—including source statistics—to enhance decoding reliability becomes increasingly important, as even modest BER improvements can translate into support for higher-order modulation schemes and thus tangible spectral efficiency gains.

One promising strategy for improving the efficiency of sources with compressible statistics combines adaptive modulation and coding (AMC) with decoding processes that incorporate prior knowledge of source statistics. AMC [4], a fundamental link adaptation method in wireless systems, continuously varies the code rate and modulation scheme according to real-time channel quality. Within this adaptive framework, leveraging prior information during decoding to achieve a lower bit error rate allows sources with non-uniform statistical properties to support more spectrally efficient, higher-order modulation formats than would be possible for equiprobable sources, leading to greater overall spectral utilization.

However, designing an effective decoding scheme under the assistance of prior information remains an open problem. Existing approaches suffer from fundamental limitations at multiple levels. The conventional method adopts a direct addition strategy [5], adding the prior log-likelihood ratio (LLR) directly to the channel LLR at initialization: $L_{init}=L_{ch}+L_{prior}$. While this Bayesian combination is theoretically optimal when prior information is perfectly accurate, the method implicitly assumes unconditional trust in the prior. In practical systems, this assumption frequently breaks down: prior probabilities derived from historical statistics contain inherent estimation errors; in time-varying scenarios, source statistics may shift faster than prior estimates can be updated; in multi-user systems, user type misclassification can lead to entirely incorrect prior assumptions. When prior information is erroneous, blindly incorporating it into the decoding process can severely degrade performance—sometimes even worse than not using prior information at all—because incorrect priors steer the decoder’s search toward wrong codeword regions.

In recent years, the integration of deep learning with channel decoding has emerged as an active research area [6,7]. Nachmani et al. [8] pioneered the Neural BP approach, transforming fixed BP iterations into a trainable neural network by assigning learnable weights to Tanner graph edges [9]. Graph neural networks (GNNs), owing to their natural compatibility with the Tanner graph structure of LDPC codes, have been applied to channel decoding with promising results [10,11]. Despite these advances, existing neural network-enhanced decoders exhibit a critical limitation in handling prior information: initial LLRs are typically computed solely from channel observations, assuming uniform bit distributions. When prior information is available, it is either ignored entirely or combined with channel information using fixed weights that cannot adapt to varying channel conditions or prior reliability. This inflexibility fundamentally limits potential performance gains in scenarios where prior information quality fluctuates [12].

Furthermore, deep neural network-based decoders face a more subtle challenge: the prior information attenuation problem. When prior information is incorporated only at the input layer, its influence progressively diminishes as it propagates through multiple network layers. Consider a standard L-layer network where prior information is used only for initialization, with subsequent layers computed recursively. The prior information continuously mixes with other signals during each layer’s transformation, causing its effective proportion in the feature representation to decay geometrically. For a five-layer network with an attenuation factor of 0.75 per layer, the prior information level at the output layer is merely about 24% of the initial level. This means that when making final bit decisions, the decoder can hardly utilize the valuable source statistical information effectively. This attenuation is particularly detrimental for LDPC decoding: on one hand, achieving good decoding performance typically requires deeper network architectures; on the other hand, deeper networks suffer more severe prior attenuation, creating an inherent tension between network depth and prior utilization.

To the best of our knowledge, no existing work has proposed a learnable mechanism that can automatically determine the trust level for prior information based on decoding context, smoothly adapt between fully reliable and completely erroneous priors, while maintaining effective prior utilization throughout the entire decoding process. This research gap constitutes the core motivation of this paper.

To address the aforementioned challenges, this paper proposes the PE-HGNN (Prior-Enhanced Heterogeneous Graph Neural Network), a novel decoder that systematically solves prior information utilization problems through three synergistic innovations. First, we propose Heterogeneous Bipartite Message Passing (HBMP), which models the Tanner graph as a heterogeneous bipartite graph that explicitly distinguishes variable nodes from check nodes. Based on the different physical meanings of the two node types, we employ differentiated activation functions and update strategies, and introduce direction-specific learnable edge weights to automatically mitigate the negative effects of short cycles. Second, we design an Adaptive Prior Fusion (APF) module that introduces a learnable gating mechanism to dynamically determine the optimal fusion ratio between channel observations and prior information. The gate values, obtained through end-to-end training, exhibit adaptive behavior consistent with Bayesian inference principles: relying more on prior information at low SNR and shifting toward channel-dominated decoding at high SNR. Third, we propose Hierarchical Prior Injection (HPI), which injects fresh prior information at every message-passing layer rather than using it only at the input layer. Each layer is equipped with an independent prior encoder, establishing direct connection paths from raw priors to each layer, fundamentally solving the prior information attenuation problem.

The main contributions of this paper are summarized as follows:

We propose a heterogeneous graph neural network architecture for LDPC decoding that explicitly distinguishes variable nodes from check nodes, employing type-specific activation functions and learnable edge weights to better capture the asymmetric roles of different node types in the decoding process.

We develop two complementary mechanisms to address the limitations of existing prior-aided decoding: an Adaptive Prior Fusion module that learns to balance channel observations and prior information based on their relative reliability, and a Hierarchical Prior Injection strategy that preserves prior information across network layers.

We validate the proposed method on standard LDPC codes and demonstrate its practical applicability in terms of both decoding performance and computational efficiency, showing that learned fusion strategies can outperform fixed combination rules while maintaining compatibility with existing communication frameworks.

## 2. Related Work

This section reviews research progress closely related to this work from three perspectives: the evolution of LDPC decoding algorithms, the utilization of prior information in channel decoding, and adaptive information fusion mechanisms in deep learning.

### 2.1. LDPC Decoding Algorithms

Traditional Iterative Decoding. LDPC codes were first proposed by Gallager in 1962 [13] but did not attract widespread attention until MacKay and Neal rediscovered their near-Shannon-limit performance in 1996 [14]. The Belief Propagation (BP) algorithm is the most prevalent soft-decision decoding method for LDPC codes, achieving near-MAP performance by iteratively exchanging probabilistic messages between variable nodes and check nodes on the Tanner graph [15,16]. However, BP’s check node update involves hyperbolic tangent and logarithmic operations with complexity $O(d_{c}\cdot \log d_{c})$, posing deployment challenges on resource-constrained platforms.

To reduce complexity, the Min-Sum (MS) algorithm [17] approximates check node updates using minimum and sign operations, reducing complexity to $O(d_{c})$ at the cost of 0.5–1.0 dB performance loss. Normalized Min-Sum (NMS) and Offset Min-Sum (OMS) [17,18] introduce correction factors to partially recover this loss while maintaining low complexity. These algorithms have become mainstream in practical implementations and are widely adopted in 5G NR [19], IEEE 802.16 [20], DVB-S2 [21], and CCSDS [22].

Despite their success, these algorithms share a fundamental limitation: update rules are fixed at the design time and cannot adapt to varying channel conditions or exploit the statistical properties of transmitted data.

Neural Network Enhanced Decoders. Nachmani et al. [8] pioneered the Neural BP approach, transforming fixed BP iterations into a trainable network by assigning learnable weights to Tanner graph edges:

(1) $$L_{v\to c}^{\left(t\right)}=L_{ch,v}+\sum_{c^{\prime }\in (v)\setminus c}w_{c^{\prime }\to v}^{\left(t\right)}\cdot L_{c^{\prime }\to v}^{\left(t-1\right)}$$

This achieved significant gains in short block length scenarios. Subsequent works extended this idea: Lugosch and Gross [23] proposed Neural OMS learning additive offsets; Several early works explored deep learning-based decoding [24], signal detection [25], and physical layer design [26]; RNN structures enabled weight sharing across iterations; Model-driven approaches [27,28] combine domain knowledge with deep learning;GNN-based decoders learned neighborhood aggregation through graph convolutions;GNN-based approaches have also been applied to polar code construction [29]; and ECCT [30] employed attention mechanisms for long-range dependencies.

More recently, Wang et al. [31] proposed ENGNN, a general edge-update-empowered GNN architecture for radio resource management in wireless networks. ENGNN introduces a full edge-update mechanism where edge representations are iteratively refined through neighbor aggregation from two categories of neighboring edges via TX-node and RX-node connections, enabling the GNN to output edge variables (e.g., beamforming vectors) with the permutation equivariance (PE) property. While ENGNN addresses heterogeneous bipartite graph modeling in wireless communications, it targets a fundamentally different problem domain (radio resource management) and does not involve iterative decoding or multi-source information fusion. In contrast, our work focuses on channel decoding with prior information, where the graph semantics (variable nodes representing coded bits, check nodes representing parity-check constraints) and the core challenge (reliability-aware fusion of heterogeneous information sources) are distinct from the beamforming and power allocation problems addressed by ENGNN. Specifically, our edge weights serve to mitigate short-cycle effects in iterative decoding on sparse factor graphs, rather than functioning as output optimization variables. The detailed comparison is provided in Table 1. Heterogeneous GNNs have also been applied to power allocation in multi-cell systems [32].

However, existing neural decoders assume uniform bit distributions, failing to account for non-equiprobable sources prevalent in practice. When prior information is available, these methods either ignore it or combine it with channel information using fixed strategies, lacking adaptive adjustment based on prior reliability.

### 2.2. Prior Information in Channel Decoding

Sources and Value. Prior statistical information about transmitted bits may originate from various scenarios: entropy-coded streams in JSCC systems exhibit non-uniform distributions; different traffic types lead to differentiated user statistics in multi-user systems; and soft information from equalizers or detectors serves as prior input in iterative receivers [33,34]. From an information-theoretic view, non-uniform sources have lower entropy than uniform sources [35], implying exploitable redundancy.

Traditional Fusion and Limitations. The conventional approach directly adds the prior LLR to channel LLR:

(2) $$L_{init}=L_{ch}+L_{prior},L_{prior}=\log\frac{P(x=0)}{P(x=1)}$$

While optimal under perfect prior accuracy, this method implicitly assumes unconditional trust in prior information. In practice, this assumption often fails: source statistics may change faster than prior estimates update; finite-sample estimation incurs inherent bias; and user misclassification leads to entirely wrong priors. When prior information is inaccurate, blindly incorporating it directs the decoder toward incorrect codeword regions, degrading performance. Table 2 summarizes the existing information fusion strategies and their respective limitations.

### 2.3. Adaptive Information Fusion in Deep Learning

Gating mechanisms have been widely validated for controlling information flow in deep learning. LSTM networks [36] regulate temporal information through input, forget, and output gates, learning which information to retain or discard. GRU [37] achieves similar functionality with a simplified structure. Attention mechanisms [38] can be viewed as soft gating—dynamically assessing input relevance and importance. GNN-based adaptive methods have also been explored for resource allocation [39]. In multi-modal fusion [40], information from different modalities is adaptively combined based on contextual reliability, sharing profound similarities with prior information fusion.

Inspired by these advances, we introduce learnable gating into neural decoders for prior fusion. Unlike temporal gating in LSTM/GRU, our prior gate determines fusion weights based on the prior strength and channel conditions jointly, enabling the decoder to rely more on priors at low SNR while shifting toward channel-dominated decoding at high SNR.

## 3. System Model and Problem Formulation

**Figure 1:**
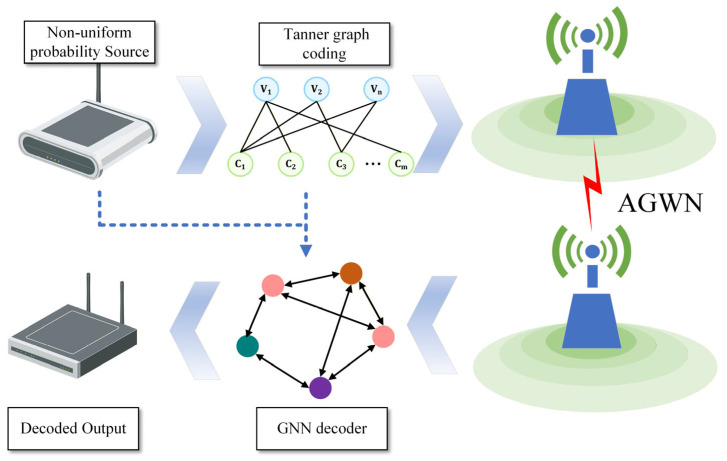
Block diagram of the LDPC-coded communication system with non-equiprobable source over AWGN channel.

The overall system architecture is illustrated in Figure 1. The source generates non-equiprobable binary symbols which are encoded using an LDPC encoder. The coded bits are then modulated using BPSK and transmitted over an AWGN channel. At the receiver, the BPSK demodulator computes the channel LLR based on the received noisy signal, while the prior LLR is derived from the source statistics. Both the channel LLR and prior LLR are utilized by the decoder to recover the transmitted information.

### 3.1. Channel Model

We consider a coded communication system employing LDPC codes over an Additive White Gaussian Noise (AWGN) channel with Binary Phase Shift Keying (BPSK) modulation. For a codeword $u=[u_{1},u_{2},\ldots ,u_{n}]\in \{0,1{\}}^{n}$, each bit $u_{i}$ is mapped to a transmitted symbol $s_{i}=1-2u_{i}$. The received signal is given by:

(3) $$y_{i}=s_{i}+n_{i}$$

where $n_{i}∼N(0,\sigma^{2})$ denotes Additive White Gaussian Noise with variance $\sigma^{2}$. The channel log-likelihood ratio (LLR), representing the reliability of the received observation, is computed as:

(4) $$L_{ch,i}=\log\frac{P(y_{i}|u_{i}=0)}{P(y_{i}|u_{i}=1)}=\frac{2y_{i}}{\sigma^{2}}$$

**Figure 2:**
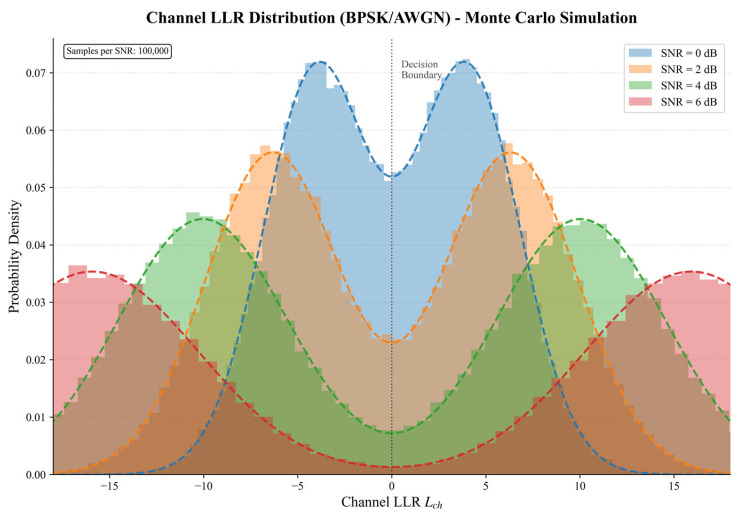
Channel LLR distribution under BPSK modulation over AWGN channel at various SNR levels.

To provide an intuitive understanding of channel observations, Figure 2 illustrates the probability density function of the channel LLR at different SNR levels. The distribution exhibits a characteristic bimodal structure: the left mode ($L_{ch}$ < 0) corresponds to transmitted bit 1, while the right mode ($L_{ch}$ > 0) corresponds to transmitted bit 0.

A key observation is the SNR-dependent behavior of these distributions. At low SNR (e.g., 0 dB), the two modes overlap significantly around the decision boundary $L_{ch}$ = 0, indicating high uncertainty in bit decisions. As SNR increases, the modes separate further apart with a reduced overlap, leading to more reliable observations. This phenomenon has important implications for decoder design: at low SNR, channel observations alone may be insufficient for accurate decoding, motivating the incorporation of prior information to assist the decision process. However, blindly trusting prior information regardless of channel quality is suboptimal, which motivates our adaptive fusion mechanism presented in Section 4.2.

### 3.2. Non-Equiprobable Source Model

We denote the prior LLR as $L_{src}$ (equivalent to $L_{prior}$ in the general literature) to emphasize its origin from source statistics. In contrast to conventional decoding that assumes uniformly distributed source bits, we consider scenarios where the source exhibits non-uniform statistics characterized by a prior probability $p_{src}=P(u_{i}=1)$. The prior LLR encoding this statistical knowledge is defined as:

(5) $$L_{src,i}=\log\frac{P(u_{i}=0)}{P(u_{i}=1)}=\log\frac{1-p_{src}}{p_{src}}$$

For systematic LDPC codes, the codeword u = [m | p] consists of k systematic bits (directly corresponding to information bits) and (n − k) parity bits generated by the encoding process. Since only the systematic bits carry direct source statistics, the prior LLR is assigned exclusively to the first k bit positions, while parity bit positions receive a prior LLR of zero:

(6) $$L_{src,i}=\left\{\begin{array}{ll} \log\frac{1-p_{src}}{p_{src}}, & i\leq k(systematic\;bits)\\ 0, & i>k(parity\;bits) \end{array}\right.$$

## 4. Proposed PE-HGNN Decoder

This section presents the Prior-Enhanced Heterogeneous Graph Neural Network (PE-HGNN) decoder in detail. We first provide an overview of the overall architecture, then elaborate on each component: the Adaptive Prior Fusion module, Heterogeneous Bipartite Message Passing layers, and Hierarchical Prior Injection mechanism.

### 4.1. Overall Architecture

The key insight behind the PE-HGNN is that effective prior utilization requires attention at multiple stages of the decoding pipeline—not just at initialization. Simply adding prior LLRs to channel LLRs, as is done conventionally, offers no mechanism to handle unreliable priors and allows prior influence to fade as information propagates through the network.

We address this by rethinking how prior information enters and flows through the decoder. At the input stage, Adaptive Prior Fusion learns to balance channel and prior information based on their relative reliability, replacing fixed combination rules with a data-driven gating mechanism. To prevent this fused signal from diminishing in deeper layers, Hierarchical Prior Injection supplies fresh prior features at each message-passing layer. The underlying message-passing structure, Heterogeneous Bipartite Message Passing, respects the distinct roles of variable and check nodes through type-specific operations and learnable edge weights.

It is important to note that the APF and HPI modules operate at different stages with complementary purposes. The APF module operates only at the input stage (before layer 0) to produce the initial variable node features $H_{v}(0)$ through reliability-aware gated fusion. It determines the initial balance between channel and prior information. The HPI module, in contrast, operates inside each subsequent message-passing layer (layers 1 through L), injecting fresh prior features into the variable node update at every layer. In the current design, HPI injects prior features with a fixed (non-gated) strength at each layer: each layer has an independent encoder (with its own parameters $W_{pr}(l)$, $b_{pr}(l)$ but does not have its own gating mechanism. The rationale for this design is to maintain a clear separation of responsibilities: APF handles the reliability-aware weighting between channel and prior information at initialization, while HPI handles the sustained availability of prior information across layers. Although HPI lacks explicit per-layer gating, the learned weight matrix $W_{v}$ in the variable node update implicitly regulates how much weight is given to the prior encoding $h_{pr}(l)$ relative to other inputs, providing some degree of adaptive prior weighting at each layer.

The decoder takes channel LLRs $L_{ch}$ and prior LLRs $L_{src}$ as input. APF produces initial variable node features, which are then refined through L layers of HBMP with HPI-injected priors. A final linear layer with sigmoid activation yields bit probability estimates.

### 4.2. Heterogeneous Bipartite Message Passing

Traditional GNN-based decoders treat the Tanner graph as a homogeneous graph, applying identical operations to all nodes. However, variable nodes (V-nodes) and check nodes (C-nodes) play fundamentally different roles: V-nodes represent coded bits carrying confidence about bit values, while C-nodes represent parity-check constraints conveying structural information. We propose Heterogeneous Bipartite Message Passing that explicitly models these differences through type-specific operations.

#### 4.2.1. Heterogeneous Graph Modeling

We model the Tanner graph as a heterogeneous bipartite graph G=(V,C,E) with two node types and two edge types. The node sets comprise variable nodes $V=v_{1},v_{2},\ldots ,v_{n}$ and check nodes $C=c_{1},c_{2},\ldots ,c_{m}$. The edge set E is determined by the parity-check matrix H: an edge exists between $v_{i}$ and $c_{j}$ if and only if $H_{ji}=1$.

**Figure 3:**
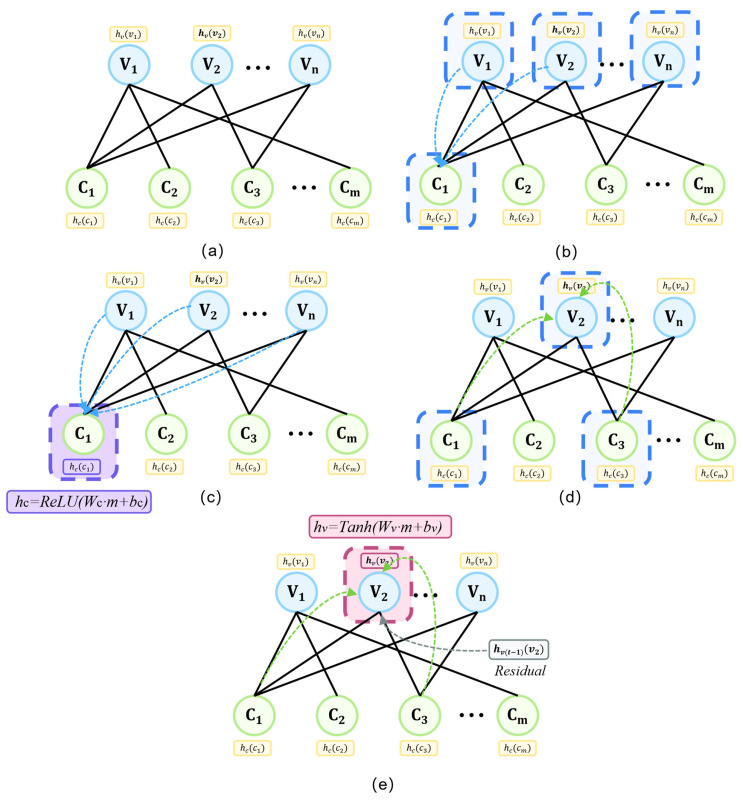
Heterogeneous Bipartite Message Passing (HBMP) process. (**a**) Heterogeneous bipartite graph modeling; (**b**) V → C message passing; (**c**) check node update; (**d**) C → V message passing; (**e**) variable node update with residual connection.

As shown in Figure 3a, V-nodes and C-nodes form a bipartite structure, where each node maintains a d-dimensional feature vector. We explicitly distinguish two directed edge types: V→C edges carry messages from variable nodes to check nodes, conveying bit reliability information; C→V edges carry messages in the reverse direction, conveying constraint feedback. Each edge type is equipped with independent learnable weights, and the two node types employ different update functions and activation functions.

#### 4.2.2. V→C Message Passing and Check Node Update

The first phase of each layer is V→C information propagation. As illustrated in Figure 3b, all V-nodes are selected as message senders. For check node $c_{j}$, let $N(c_{j})=\{v_{i}:H_{ji}=1\}$ denote its neighboring variable nodes. Message aggregation employs weighted summation:

(7) $$m_{v\to c_{j}}=\sum_{v_{i}\in N(c_{j})}w_{ij}^{vc}\cdot h_{v_{i}}$$

where $w_{ij}^{vc}$ denotes the learnable V→C edge weight and $h_{v_{i}}$ is the current feature of variable node $v_{i}$.

As shown in Figure 3c, taking check node $C_{1}$ as an example, it receives messages from all its neighboring V-nodes. The aggregated message is concatenated with its own features, followed by linear transformation and ReLU activation:

(8) $${h^{\prime }}_{c_{j}}=ReLU(W_{c}\cdot [h_{c_{j}};m_{v\to c_{j}}]+b_{c})$$

where $W_{c}\in R^{d\times 2d}\;and\;b_{c}\in R^{d}$ are learnable parameters. ReLU is chosen because check nodes process reliability metrics that should be non-negative, and its sparsity-inducing property facilitates the learning of discriminative representations.

#### 4.2.3. C→V Message Passing and Variable Node Update

The second phase is C→V information propagation. As illustrated in Figure 3d, the updated C-nodes are now selected as message senders. For variable node $v_{i}$, let $N(v_{i})=\{c_{j}:H_{ji}=1\}$ denote its neighboring check nodes. Message aggregation is similarly computed as:

(9) $$m_{c\to v_{i}}=\sum_{c_{j}\in N(v_{i})}w_{ji}^{cv}\cdot {h^{\prime }}_{c_{j}}$$

where $w_{ji}^{cv}$ denotes the learnable C→V edge weight, independent of $w_{ij}^{vc}$.

As shown in Figure 3e, taking variable node $V_{2}$ as an example, it receives messages from its neighboring C-nodes. The variable node update integrates three information sources: its own features, aggregated check messages, and layer-wise prior injection (detailed in Section 4.4):

(10) $${h^{\prime }}_{v_{i}}=Tanh(W_{v}\cdot [h_{v_{i}};m_{c\to v_{i}};h_{pr}^{\left(l\right)}]+b_{v})$$

where $h_{pr}^{\left(l\right)}$ is the prior encoding at layer l, and $W_{v}\in R^{d\times 3d},\;b_{v}\in R^{d}$ are learnable parameters. Tanh activation is chosen because variable nodes carry LLR-like information where positive values indicate preference for bit 0 and negative values for bit 1; Tanh preserves this bipolar characteristic while providing numerical stability with output range (−1, 1).

Additionally, as indicated by the gray dashed arrow in Figure 3e, a residual connection from the previous layer is added to facilitate gradient flow:

(11) $$h_{v_{i}}^{\left(l\right)}={h^{\prime }}_{v_{i}}+h_{v_{i}}^{\left(l-1\right)}$$

This cumulative update mirrors the iterative refinement in traditional BP, where each iteration performs incremental correction based on previous estimates.

#### 4.2.4. Learnable Edge Weights

A key design of HBMP is the learnable edge weights $\left\{w_{ij}^{vc}\right\}$ and $\left\{w_{ji}^{cv}\right\}$. The Tanner graphs of LDPC codes typically contain short cycles (e.g., length-4 or length-6 cycles), which cause message correlation and degrade BP performance. Learnable weights enable the network to automatically identify and down-weight edges involved in short cycles. Additionally, since different edges contribute unequally to decoding, the network can learn to emphasize informative connections. The independence of V→C and C→V weights allows asymmetric message propagation strengths, a flexibility unavailable in traditional BP. In implementation, edge weights are initialized to 1.0 and transformed through softplus to ensure positivity.

In the current implementation, the V→C edge weights $\left\{w_{ij}^{vc}\right\}$ and C→V edge weights $\left\{w_{ji}^{cv}\right\}$ are shared across all L message-passing layers, while remaining independent from each other. This design is motivated by three considerations. First, parameter efficiency: for the IEEE 802.11n LDPC code (576,432) with 1152 non-zero entries in H, sharing weights across layers significantly reduces the total number of trainable parameters, which is important for avoiding overfitting. Second, the primary purpose of learnable edge weights is to mitigate the impact of short cycles in the Tanner graph, which is a structural property of the code rather than a layer-dependent feature—short cycles at a given edge cause message correlation regardless of which layer the message passing occurs in. Third, this design follows the convention established in the Neural BP literature [7], where edge weights are typically shared across iterations/layers, mirroring the structure of standard BP where the same update rules are applied at every iteration. We note that layer-wise (unshared) edge weights could potentially further improve performance at the cost of increased parameters, which we leave as a direction for future investigation.

### 4.3. Adaptive Prior Fusion Module

The Adaptive Prior Fusion (APF) module addresses a fundamental question: how should channel observations and prior information be optimally combined? Traditional approaches employ fixed weighting schemes that cannot adapt to varying channel conditions. We propose a learnable gating mechanism that automatically adjusts fusion weights based on input characteristics.

#### 4.3.1. Dual-Path Feature Encoding

**Figure 4:**
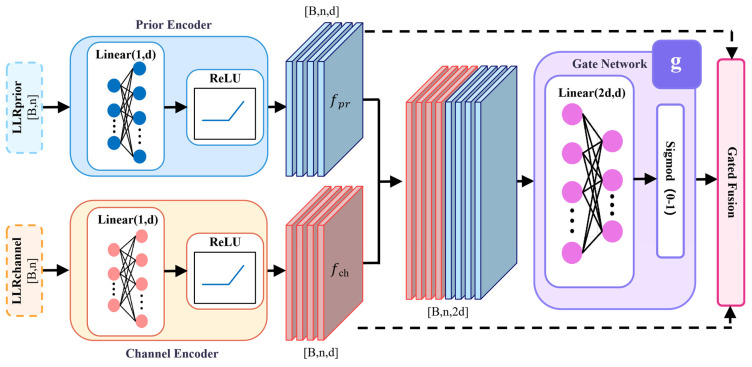
Structure of the Adaptive Prior Fusion (APF) module.

As shown in Figure 4, the channel and prior LLRs are independently encoded into a d-dimensional feature space.

The channel encoder transforms scalar channel LLRs into high-dimensional feature vectors:

(12) $$f_{ch}=ReLU(W_{ch}\cdot L_{ch}+b_{ch})$$

where $W_{ch}\in R^{d\times 1}$ and $b_{ch}\in R^{d}$ are learnable parameters, yielding $f_{ch}\in R^{n\times d}$.

Similarly, the prior encoder processes prior LLRs:

(13) $$f_{pr}=ReLU(W_{pr}\cdot L_{src}+b_{pr})$$

With independent parameters $W_{pr}\in R^{d\times 1}$ and $b_{pr}\in R^{d}$. The use of separate encoders enables the network to learn distinct optimal representations for channel and prior information.

#### 4.3.2. Gated Fusion Mechanism

The core of APF is a gating network that adaptively determines the fusion ratio between the two information sources. The encoded features are first concatenated along the feature dimension: $f_{cat}=[f_{ch};f_{pr}]\in R^{n\times 2d}$.

The concatenated features pass through a linear transformation followed by sigmoid activation to generate gate values: $g=\sigma (W_{g}\cdot f_{cat}+b_{g})$, where $W_{g}\in R^{d\times 2d}$, $b_{g}\in R^{d}$, and σ(⋅) denotes the sigmoid function. Each element of $g\in R^{n\times d}\;lies\;in\;(0,1)$, representing the relative importance of channel information at that position.

The initial variable node features are obtained through gated fusion:

(14) $$H_{v^{\left(0\right)}}=g⊙f_{ch}+(1-g)⊙f_{pr}$$

This mechanism enables the network to dynamically adjust fusion strategies based on input conditions. At low SNR, where channel observations are heavily corrupted by noise, the network tends to assign smaller gate values, relying more on prior information. Conversely, at high SNR, gate values approach unity as channel observations become sufficiently reliable. The entire fusion process is jointly trained with other network parameters through end-to-end optimization.

Why feature-based gating rather than a scalar reliability indicator? An arguably simpler alternative would be to derive the gate directly from the channel LLR magnitude $\left|L_{ch}\right|$, which already provides a scalar measure of observation reliability. We deliberately chose the feature-based design for the following reason. The scalar $\left|L_{ch}\right|$, summarizes each bit’s channel quality into a single number, but the optimal fusion strategy is not a function of channel reliability alone—it also depends on the strength and relevance of the prior. Consider two scenarios at the same moderate SNR: one with $p_{src}=0.1$ and another with $p_{src}=0.4$. A gate conditioned solely on $\left|L_{ch}\right|$ would produce identical fusion ratios in both cases, ignoring the substantial difference in prior informativeness. By contrast, our gating network receives the concatenation $\left[f_{ch};f_{pr}\right]$ and can therefore adjust its output based on the joint characteristics of both sources. The experimental results in Section 5.4, where gate values shift systematically with $p_{src}$ at fixed SNR, confirm that this joint conditioning is indeed exploited by the trained network.

Beyond this, the feature-based design offers a subtlety that scalar gating cannot: per-dimension fusion control. Since g is d-dimensional, different feature channels can adopt different fusion ratios for the same bit position. This is meaningful because the d-dimensional encoding decomposes the input LLR into multiple latent aspects—some of which may benefit more from channel evidence while others benefit more from prior knowledge. A scalar gate, by contrast, would impose a uniform ratio across all feature dimensions, discarding this degree of freedom.

Why the learned gate exhibits SNR-adaptive behavior. The monotonically increasing relationship between gate values and SNR was not built into the architecture—it is a consequence of training under the BCE loss. To understand why, note that the loss gradient with respect to the gate involves the term $\left(f_{ch,i}-f_{pr,i}\right)$, which reflects the discrepancy between channel and prior feature representations at each position. At low SNR, channel LLR magnitudes are small, so the corresponding encoded features $f_{ch}$ carry limited discriminative power. In this regime, relying more heavily on prior features reduces the bit error rate, and the gradient signal drives the gate parameters toward lower values. At high SNR, the situation reverses: $f_{ch}$ becomes highly informative, and the loss landscape favors gate values close to unity. The network thus arrives at a fusion strategy that is qualitatively consistent with Bayesian reasoning—weighting each source in proportion to its reliability—not because we imposed this behavior, but because it is the strategy that minimizes the training objective across the SNR range encountered during learning.

This emergent property has a practical implication worth noting: the decoder does not require an external SNR estimator to adapt its fusion strategy. The channel condition is implicitly encoded in $f_{ch}$ through the magnitude and distribution of its feature activations, and the gating network has learned to read this encoding. This stands in contrast to conventional approaches where prior weighting must be manually tuned for each operating point.

### 4.4. Hierarchical Prior Injection

Deep neural network-based decoders face a critical challenge: prior information decay. When prior information is incorporated only at the input layer, its influence diminishes progressively through multi-layer propagation.

HPI is specifically designed to complement the APF module. While APF determines the optimal initial balance between channel and prior information, HPI ensures that prior information remains available throughout the multi-layer decoding process. HPI is most beneficial in the low-to-medium SNR regime, where channel observations are unreliable and prior information is most valuable. In high-SNR scenarios, the decoder naturally relies less on priors—a behavior handled by the APF module.

#### 4.4.1. Prior Decay Problem

Consider a standard L-layer network where prior information $L_{src}$ is used only for initialization, i.e., $h^{\left(0\right)}=f(L_{ch},L_{src})$, with subsequent layers computed via $h^{\left(l\right)}=g(h^{\left(l-1\right)})$. Through successive transformations, the prior signal continuously mixes with other signals, causing its effective proportion in the feature representation to decay geometrically.

**Figure 5:**
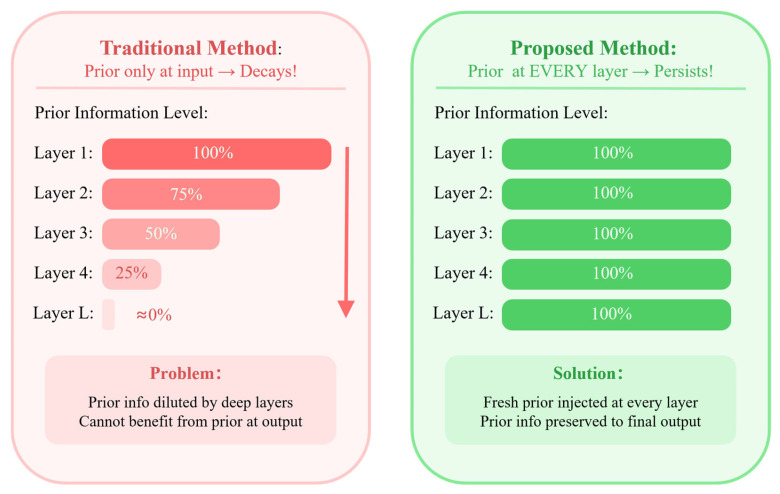
Prior information decay across network layers in conventional deep decoders.

Figure 5 provides a conceptual illustration of the prior decay mechanism. As a concrete example, a network retaining ~75% of prior signal per layer preserves only ~24% at layer 5. While the exact rate depends on architecture and training dynamics, the qualitative trend is well-established. Importantly, this mechanism is empirically validated by the ablation study in Section 5.7: removing HPI leads to noticeable BER degradation in the low-to-medium SNR regime, directly demonstrating that prior information attenuates when injected only once.

#### 4.4.2. Layer-Wise Prior Injection

**Figure 6:**
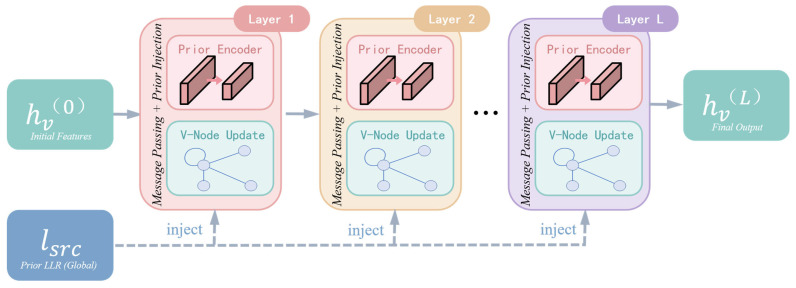
Hierarchical Prior Injection (HPI) mechanism with layer-specific prior encoders.

To address this problem, we propose injecting fresh prior information at every message-passing layer rather than using it only once at the input. As illustrated in Figure 6, each layer is equipped with an independent prior encoder that transforms the raw prior LLR into layer-specific feature representations:

(15) $$h_{pr}^{\left(l\right)}=ReLU(W_{pr}^{\left(l\right)}\cdot L_{src}+b_{pr}^{\left(l\right)})$$

where $W_{pr}^{\left(l\right)}\in R^{d\times 1}$ and $b_{pr}^{\left(l\right)}\in R^{d}$ are layer-specific learnable parameters.

Layer-specific encoders are employed rather than shared encoders because different layers process information at different abstraction levels: early layers focus on local reliability estimation, middle layers progressively integrate neighborhood information, and later layers capture more global code structure features. Layer-specific parameters allow the network to learn the most suitable prior representation for each processing stage.

Compared to residual connections, skip connections, and dense connections, HPI is more targeted: residual connections [41] pass fused features rather than raw priors; skip connections typically connect only the first and last layers; and dense connections introduce unnecessary complexity and redundancy. HPI is specifically designed for sustained prior utilization with minimal overhead, establishing direct paths from the source prior to each processing stage while allowing layer-specific adaptation through independent encoders.

### 4.5. Loss Function and Training

The network is trained using Binary Cross-Entropy (BCE) loss:

(16) $$L=-\frac{1}{Bn}\sum_{b=1}^{B}\sum_{i=1}^{n}\left[u_{i}^{\left(b\right)}{\log\hat{y}}_{i}^{\left(b\right)}+(1-u_{i}^{\left(b\right)})\log(1-{\hat{y}}_{i}^{\left(b\right)})\right]$$

where $u_{i}^{\left(b\right)}$ is the ground-truth bit and ${\hat{y}}_{i}^{\left(b\right)}$ is the predicted probability. BCE loss provides dense gradients for each bit and directly correlates with the BER metric. Implementation uses BCEWithLogitsLoss for numerical stability.

Training data is generated online with SNR sampled from {1.0,1.5,2.0,2.5,3.0,3.5,4.0} dB and $p_{src}$ sampled from {0.1,0.15,0.2,0.25,0.3,0.35,0.4}. This multi-SNR, multi-$p_{src}$ training strategy is a deliberate design choice motivated by three factors. First, in practical wireless systems, channel SNR fluctuates continuously due to user mobility, fading, and interference; a deployed decoder must perform well across the entire operating range. Second, and critically, the APF module must learn SNR-adaptive gating behavior—training at a single SNR would collapse gate values to a single operating point, defeating the purpose of adaptive fusion. Multi-SNR exposure is what enables the gate to smoothly transition from prior-dominated fusion at low SNR to channel-dominated fusion at high SNR (Figure 11). Third, multi-SNR training ensures that the learned edge weights are robust across operating conditions rather than optimized for a single noise level. We note that this approach does not significantly increase computational burden: the total number of training samples remains the same, with samples simply distributed across different SNR values through online generation. Each training batch is generated with a randomly sampled SNR, so the per-epoch cost is identical to single-SNR training. The network is trained using the Adam optimizer with initial learning rate 0.001 [42].Weight initialization follows [43]. Learning rate reduction is applied when validation BER shows no improvement for consecutive epochs. Table 3 summarizes the training configuration.

## 5. Experimental Results

This section validates the effectiveness of the proposed PE-HGNN decoder through systematic experiments. We first introduce the experimental setup, then present comprehensive evaluations from five perspectives: BER performance, impact of source statistics, interpretability of the adaptive mechanism, computational complexity, and ablation analysis.

### 5.1. Experimental Setup

#### 5.1.1. LDPC Code Parameters

Experiments are conducted on the IEEE 802.11n standard LDPC code, which employs a quasi-cyclic structure [44], which serves as a widely recognized benchmark in both academic research and industrial applications. The detailed code parameters are listed in Table 4, where the rate-3/4 configuration represents a typical high-throughput scenario in practical wireless communication systems.

**Figure 7:**
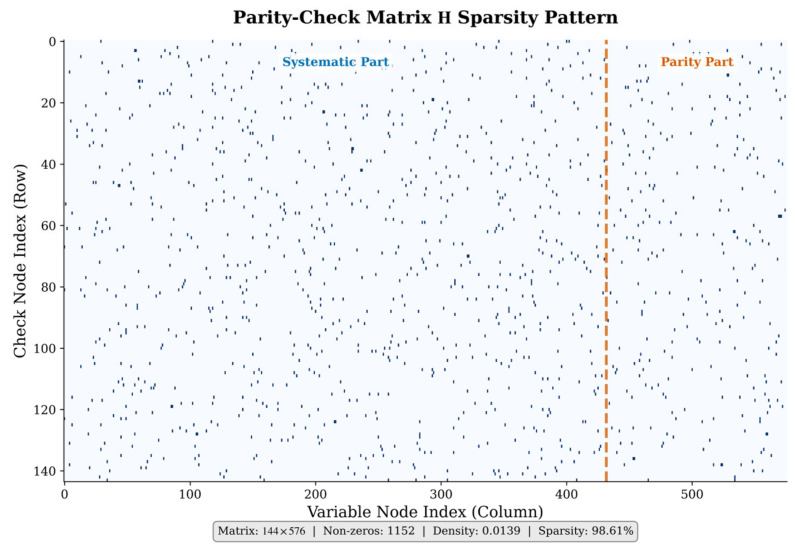
Sparsity pattern of parity-check matrix H.

Figure 7 illustrates the sparsity pattern of the parity-check matrix H for the IEEE 802.11n LDPC code (576, 432). The matrix has dimensions 144 × 576 with only 1152 non-zero entries, yielding a density of 0.0139 (sparsity 98.61%). The dashed line separates the systematic part (columns 0–431) from the parity part (columns 432–575).

#### 5.1.2. Baseline Methods

To comprehensively evaluate the performance of the PE-HGNN, we select the following four representative methods as baselines:

BP-50: Standard belief propagation algorithm with flooding schedule, maximum 50 iterations, and early termination upon parity-check satisfaction. This configuration represents the performance upper bound of traditional decoding algorithms under sufficient iterations.

BP-5: Iteration-limited belief propagation algorithm, where the iteration count matches the number of message-passing layers in the PE-HGNN, enabling fair comparison under equivalent complexity.

BP+Prior: Belief propagation decoder initialized with combined LLRs $\left(L_{init,i}=L_{ch,i}+L_{src,i}\right)$, representing the classical approach for incorporating prior information into traditional decoding frameworks.

Homo-GNN: Homogeneous graph neural network decoder that applies uniform update mechanisms to both variable and check nodes. Prior information is introduced only at the input layer, serving to validate the necessity of heterogeneous modeling. Table 5 summarizes the configurations and purposes of all baseline decoders.

All neural network-based methods are trained using identical data generation pipelines and hyperparameter configurations to ensure fair comparison.

#### 5.1.3. Network Depth Selection

The number of message-passing layers L = 5 is chosen based on three considerations. (1) Fair comparison: each GNN layer corresponds structurally to one BP iteration, so L = 5 matches BP-5 in computational depth, ensuring gains are attributable to architectural innovations rather than extra computation. (2) Information-theoretic sufficiency: with L = 5, the propagation radius reaches 10 hops in the bipartite Tanner graph, substantially exceeding the girth of 6, ensuring each variable node receives constraint information from a sufficiently large neighborhood. (3) Consistency with the Neural BP literature [8], where 5 iterations of weighted BP are established as sufficient for short-to-medium block length codes.

#### 5.1.4. Evaluation Metrics

We adopt bit error rate (BER) and coding gain as performance metrics. BER is defined as the bit-level discrepancy rate between estimated and true codewords:

(17) $$BER=\frac{1}{n\cdot N_{test}}\sum_{j=1}^{N_{test}}\sum_{i=1}^{n}1\left[{\hat{u}}_{i}^{\left(j\right)}\neq u_{i}^{\left(j\right)}\right]$$

Coding gain quantifies the SNR savings (in dB) required to achieve a target BER level.

### 5.2. BER Performance Analysis

**Figure 8:**
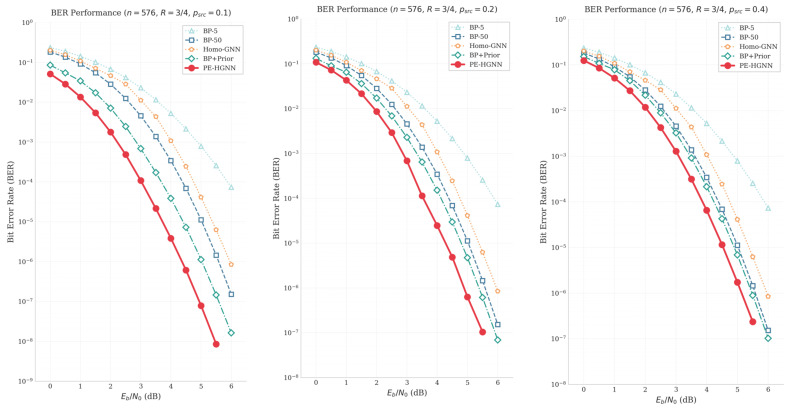
BER performance comparison under *p_src_* = 0.1, 0.2, 0.4.

**Figure 9:**
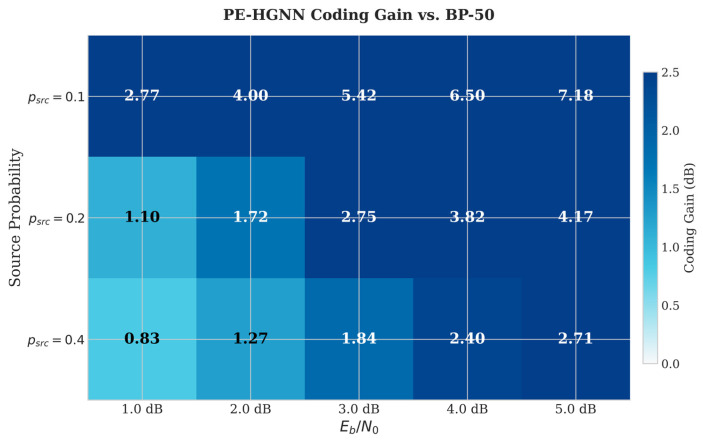
Coding gain of PE-HGNN relative to BP-50 across different SNR and $p_{src}$ values.

Figure 8 presents the BER performance curves for source probabilities $p_{src}\in \{0.1,0.2,0.4\}$, while Figure 9 visualizes the coding gain of the PE-HGNN relative to BP-50 in heatmap form.

From an information-theoretic perspective, the source probability $p_{src}$ participates in the decoding process through the prior LLR $L_{src}=\ln\frac{1-p_{src}}{p_{src}}.$ As $p_{src}$ deviates from 0.5, $\left|L_{prior}\right|$ increases correspondingly, providing stronger soft-decision constraints for the decoder. The experimental results align well with this theoretical expectation.

As the source probability p deviates from 0.5, the prior LLR provides additional soft information that shifts the waterfall region toward lower SNR values. This phenomenon can be interpreted as the prior information effectively reducing the equivalent channel noise, thereby enabling the decoder to enter the steep error-correction regime at lower SNR levels.

As expected, BP-5, BP-50, and Homo-GNN show similar BER performance regardless of $p_{src}$, since none of these methods incorporate prior information into the decoding process. When prior information is utilized, both BP+Prior and the PE-HGNN benefit from stronger priors (i.e., larger |$L_{prior}$|). The heatmap further shows that the PE-HGNN achieves more pronounced gains under favorable source conditions ($p_{src}$ = 0.1), while the improvement becomes marginal as $p_{src}$ approaches 0.4. Across all tested configurations, the PE-HGNN outperforms BP+Prior. We attribute this improvement to two factors: the heterogeneous graph structure better captures the distinct roles of variable and check nodes during message passing, and the APF module learns to adjust fusion weights according to input conditions rather than relying on fixed combination rules.

### 5.3. Impact of Source Statistics

**Figure 10:**
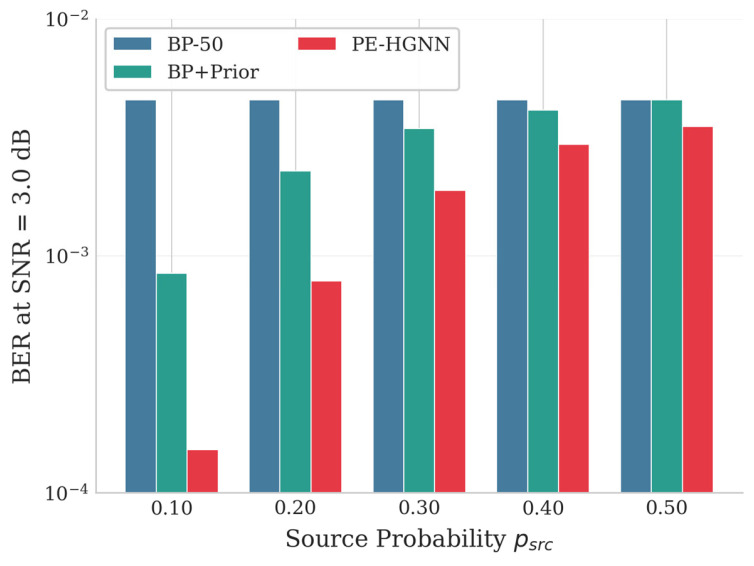
Impact of source statistics. BER at SNR = 3.0 dB.

To systematically investigate the influence of source statistics on decoding performance, we evaluate all methods across $p_{src}\in 0.10,0.20,0.30,0.40,0.50.$ Figure 10 shows BER performance at SNR = 3.0 dB.

The results show a clear trend: as $p_{src}$ decreases from 0.50 to 0.10, the coding gain of the PE-HGNN over BP-50 grows from 0.24 dB to 1.85 dB. This is expected from an information-theoretic standpoint, since lower $p_{src}$ values correspond to stronger prior information that can be leveraged during decoding. Comparing the PE-HGNN with BP+Prior, we observe that the PE-HGNN consistently achieves better performance across all $p_{src}$ settings, and the gap becomes more significant as the prior strength increases—up to 0.72 dB at $p_{src}$ = 0.10. This suggests that Hierarchical Prior Injection is more effective than simply adding prior LLRs at initialization.

It is also worth noting that when $p_{src}$ = 0.50 (i.e., $L_{prior}$ = 0), the PE-HGNN still outperforms BP-50 by 0.24 dB. Since no prior information is available in this case, the gain comes solely from the learned edge weights. This result indicates that the PE-HGNN remains effective even without prior information, which is important for practical deployment where source statistics may not always be available.

### 5.4. Interpretability Analysis of Adaptive Mechanism

**Figure 11:**
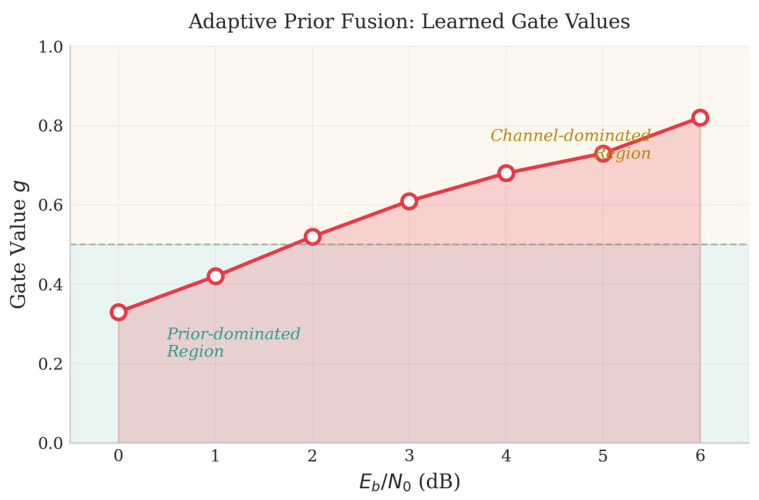
Learned gate values as a function of SNR.

To understand how the APF module works, we examine the learned gate values g under different SNR conditions (Figure 11).

Figure 11 presents gate values obtained with $p_{src}$ = 0.2 as the representative case. The gate g increases smoothly with SNR: at low SNR (0–2 dB), the average stays below 0.5 (ġ ≈ 0.36–0.52), shifting to 0.70–0.78 at high SNR. The crossover point near SNR ≈ 2 dB indicates equal contribution from both sources.

What makes this result interesting is that such behavior was not explicitly programmed—it emerges naturally from end-to-end training. The network learns on its own to rely more on priors when channel quality is poor and to trust channel observations when they are reliable, which aligns well with Bayesian reasoning. This provides some evidence that the learned fusion strategy is not arbitrary but follows a principled trade-off. The above analysis demonstrates that the APF module learns to adjust fusion weights based on SNR. A natural question arises: can this adaptive mechanism also handle situations where the prior information itself is inaccurate? We address this question in the next subsection.

### 5.5. Robustness Against Prior Mismatch

**Figure 12:**
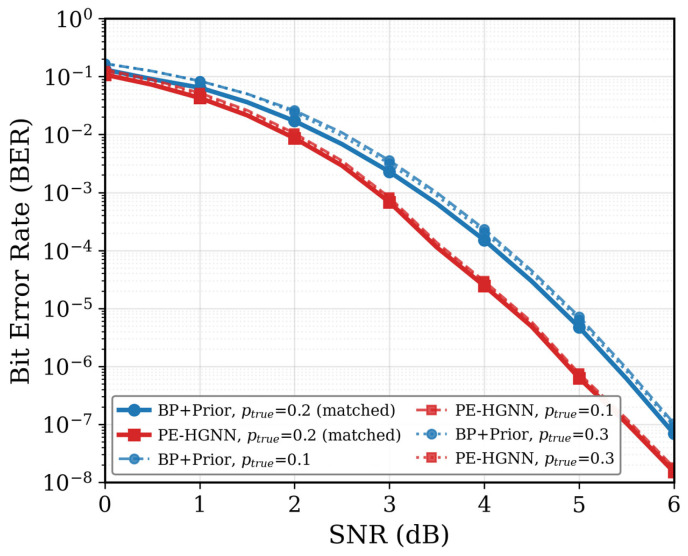
BER performance under prior mismatch.

Figure 12 BER performance under prior mismatch. In practice, the assumed prior probability may differ from the true source statistics due to estimation errors or distribution drift. Here we train both decoders with $p_{assumed}$ = 0.2 and test them under $p_{ture}$ = 0.1, 0.2, and 0.3. When $p_{ture}$ = 0.2 (solid lines), both methods perform as expected. Under mismatch (dashed and dotted lines), BP+Prior shows notable degradation since it blindly trusts the incorrect prior. In contrast, the PE-HGNN is less affected, suggesting that the learned gating mechanism helps reduce reliance on unreliable priors.

**Figure 13:**
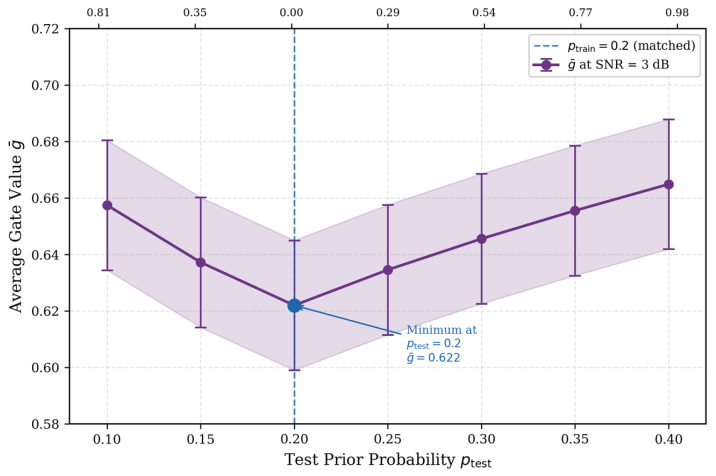
Average gate value $\bar{g}$ of the APF module as a function of $p_{test}$ at SNR = 3 dB (model trained with $p_{train}=0.2$). The U-shaped curve with its minimum at the matched condition demonstrates that the learned gate automatically increases channel reliance under prior mismatch.

To provide direct mechanistic evidence for this observation, we further measure the average gate value g of the APF module as a function of the test prior probability $p_{test}$, with the model fixed at $p_{train}=0.2$ and SNR fixed at 3 dB. Figure 13 presents the results across $p_{test}\in \{0.10,0.15,0.20,0.25,0.30,0.35,0.40\}$. The secondary x-axis reports the corresponding LLR deviation $\left|\Delta \lambda |=|\lambda (p_{test})-\lambda (p_{train})\right|$, which quantifies the magnitude of prior mismatch in the log-likelihood ratio domain.

The results reveal a clear U-shaped pattern: the average gate value reaches its minimum at the matched condition and increases monotonically as the mismatch grows in either direction, reaching $\bar{g}\approx 0.659\;at{\;p}_{test}=0.10(|\Delta \lambda |\;=0.81)\;and\bar{\;g}\approx 0.664\;at\;p_{test}\;=0.40(|\Delta \lambda |\;=0.98).$ Since g represents the weight assigned to channel observations, a higher g under mismatch directly implies that the APF module automatically reduces its reliance on the unreliable prior and compensates by increasing the contribution of channel observations—without any explicit supervision for this behavior.

This adaptive response can be understood through the structure of the gating network. The gate receives as input the concatenation of channel-encoded features $f_{ch}$ and prior-encoded features $f_{pr}$. When $p_{test}$ deviates from $p_{train}$, the prior LLRs shift accordingly, causing $f_{pr}$ to become statistically inconsistent with the prior representations the gating network has learned to associate with reliable information. This inconsistency propagates through the sigmoid gating projection, driving the output gate values upward and thereby increasing the relative weight of the more internally consistent channel features. Importantly, this self-protective behavior emerges naturally from end-to-end training under the BCE loss across a broad distribution of SNR values and source probabilities—it was not explicitly engineered. The gate value responds primarily to the magnitude of |Δλ| rather than the direction of mismatch, as evidenced by the near-identical g values observed at comparable |Δλ| levels on both sides of the matched condition, which is consistent with a principled reliability-aware fusion strategy. This direct evidence provides a quantitative mechanistic explanation for the BER robustness observed in Figure 12.

### 5.6. Computational Complexity Analysis

**Figure 14:**
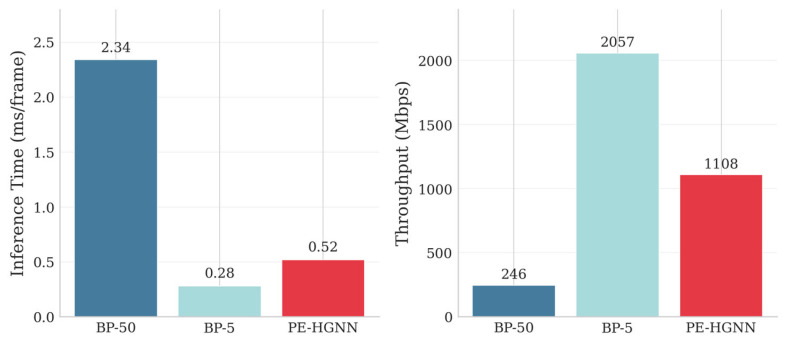
Computational complexity comparison.

Figure 14 compares the computational efficiency of different methods in terms of inference latency and decoding throughput, with all measurements conducted on an NVIDIA RTX 5090 GPU platform.

The PE-HGNN achieves a per-frame inference latency of 0.52 ms, representing a 4.5× speedup over BP-50, primarily attributable to the highly parallelizable nature of graph neural network operations on GPU architectures. In terms of throughput, the PE-HGNN reaches 1108 Mbps, a 4.5× improvement over BP-50. Although BP-5 achieves the highest throughput (2057 Mbps) due to its minimal iteration count, it suffers significant BER degradation.

We analyze per-component FLOPs (each MAC = 2 FLOPs) for the configuration in Table 3 (n = 576, m = 144, |E| = 1152, d = 64, L = 5).

The APF module (executed once) costs 4nd^2^ + 13nd FLOPs, dominated by the gating projection $W_{g}$ ∈ $R^{\left\{d\times 2d\right\}}$. Each HBMP layer costs 6nd^2^ + 4md^2^ + 4|E|d + 5nd + 2md FLOPs, where the variable node update ($W_{v}$ ∈ $R^{\left\{d\times 3d\right\}}$) is the primary contributor. Each HPI encoder adds only 4nd FLOPs. The overall asymptotic complexity is O(L(n+m)d^2^ + L$\left|E\right|$d).The detailed FLOPs and parameter breakdown is provided in Table 6.

HBMP layers dominate total cost (~89%), while HPI adds less than 1% overhead, confirming that sustained prior injection is computationally negligible. Although the PE-HGNN’s total FLOPs (~95.8 M) exceed BP-50’s (~0.92 M) by two orders of magnitude, BP relies on sequential scalar iterations with transcendental functions, whereas the PE-HGNN performs parallel d-dimensional matrix operations with high GPU utilization—explaining the 4.5× wall-clock speedup in Figure 14.

### 5.7. Ablation Study

**Figure 15:**
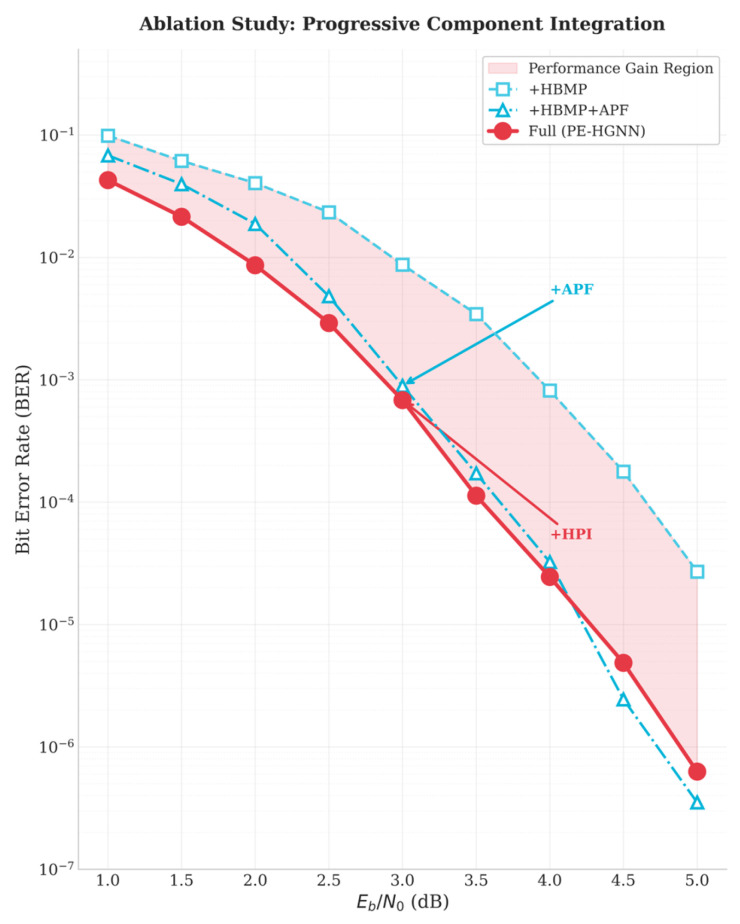
Ablation study: BER performance of different module combinations.

To quantify the contribution of each component, we conducted ablation experiments by progressively introducing modules. Figure 15 presents the performance of different module combinations across SNR conditions in a heatmap form.

#### 5.7.1. Analysis of Module Contributions

The ablation results reveal that each module’s contribution varies with SNR, reflecting the shifting balance between channel and prior information across operating conditions.

In the low-to-medium SNR range (1.0–4.0 dB), the full PE-HGNN consistently achieves the lowest BER, and removing HPI causes measurable degradation. This degradation itself serves as empirical evidence for the prior decay problem illustrated in Figure 5: were prior information fully preserved through network layers without HPI, its removal would produce no performance difference. The fact that it does confirms that single-injection prior features attenuate during propagation. At high SNR (≥4.5 dB), however, the model without HPI performs marginally better—a point we discuss below.

Figure 15 presents four progressive configurations to isolate each module’s contribution. Their respective gains have distinct origins.

HBMP gain (Base → +HBMP). Replacing homogeneous node updates with heterogeneous ones yields improvement across all SNR levels, independent of prior information. The gain stems from matching activation functions to physical node semantics are: ReLU for check nodes, whose outputs represent non-negative reliability metrics, and Tanh for variable nodes, whose outputs carry bipolar LLR-like sign information. Additionally, independent V→C and C→V edge weights permit asymmetric message damping that helps suppress short-cycle interference—a structural property that benefits decoding uniformly regardless of noise level.

APF gain (+HBMP → +APF). Introducing Adaptive Prior Fusion brings further improvement that, as Figure 15 shows, grows with SNR (~0.15 dB at 1.0 dB SNR to ~1.4 dB at high SNR). At first glance this trend seems counterintuitive, since prior information should be most valuable when channel quality is poor. The explanation lies in the decoder’s internal state: at very low SNR, the message-passing layers operate on heavily corrupted features, limiting the decoder’s capacity to meaningfully integrate any additional information source. As SNR increases, the backbone produces progressively more coherent feature representations, providing a reliable substrate upon which the gating mechanism can effectively blend prior knowledge. In other words, effective fusion requires that at least one source carries sufficient quality—a condition increasingly satisfied at higher SNR.

HPI gain (+APF → Full). The additional gain from Hierarchical Prior Injection concentrates in the 1.0–4.0 dB range, precisely the regime where channel observations are unreliable and the decoder depends on prior guidance throughout the iterative process. Without HPI, prior influence fades layer by layer; with HPI, each layer receives a fresh prior encoding, sustaining guidance where it matters most. Beyond 4.5 dB, this injection becomes mildly counterproductive: channel-derived features at that point already carry strong decision confidence, and continued prior injection introduces competing signals that can slightly perturb convergence. This observation motivates the adaptive injection strategy discussed in Section 5.7.3.

**Figure 16:**
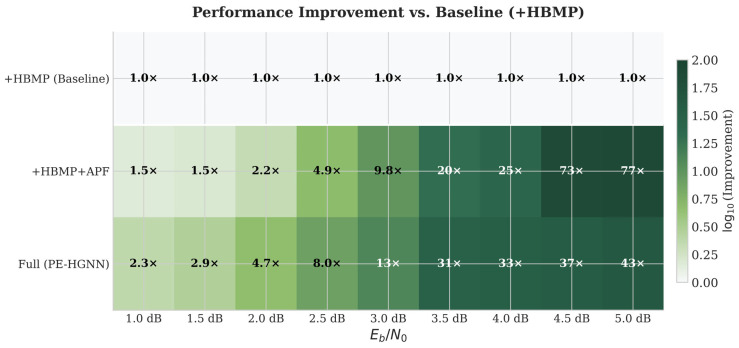
Marginal gains of APF and HPI modules versus SNR.

The complementarity between APF and HPI is evident from Figure 16: APF’s marginal gain rises monotonically with SNR while HPI’s marginal gain peaks in the low-to-medium range, with maximum synergy occurring around 2.5–4.0 dB. Together, the two modules cover the full operating range without mutual interference.

This asymmetry leads to the following consequence: in the high SNR region, channel LLR magnitudes increase substantially ($|L_{ch}|\propto SNR$), and variable node confidences become sufficient for reliable decisions. At this point, the HPI module continues injecting prior encodings $h_{pr}^{\left(l\right)}$ at a fixed strength, causing these prior signals to compete with high-confidence channel information in the feature space. From an optimization perspective, the iterative belief propagation process can be viewed as a search in codeword space. High-quality channel LLRs have already narrowed the search to the neighborhood of the correct codeword, while continuously injected prior information introduces additional “soft constraints.” Although these constraints are statistically correct, they may deviate from the specific codeword direction indicated by the current received signal, thereby perturbing the convergence trajectory.

More specifically, consider the feature fusion in variable node updates: $h_{\prime v_{i}}=Tanh(W_{v}\cdot [h_{v_{i}};m_{c}\to v_{i};h_{pr}^{\left(l\right)}]+b_{v})$. At high SNR, $hv_{i}$ and $m_{c}\to v_{i}$ already contain strong decision tendencies, while the inclusion of $h_{pr}^{\left(l\right)}$ dilutes the purity of these tendencies. Although network weights $W_{v}$ can learn moderate suppression of prior components during training, the network cannot achieve complete shielding of prior components for high SNR scenarios because training data covers a broad SNR range.

In contrast to HPI’s “unconditional injection” strategy, the APF module exhibits fundamentally different behavior. The key lies in the input-dependent nature of gate values $g:g=\sigma (W_{g}\cdot [f_{ch};f_{pr}]+b_{g})$. The gating network takes channel-encoded features $f_{ch}$ as one of its inputs, and the magnitude of fch directly reflects channel observation reliability. At high SNR, $\left|f_{ch}\right|$ increases, and the gating network infers that channel information is more trustworthy, outputting higher g values to increase channel weight and reduce prior weight. This mechanism endows APF with intrinsic SNR-awareness, enabling adaptive adjustment of fusion strategies without explicit SNR estimation. The gate value curves in Section 5.4 corroborate this analysis: $\bar{g}$ stabilizes at 0.70–0.78 in the high SNR region, meaning APF proactively compresses prior contribution to 22–30%, effectively avoiding interference from prior information on high-quality channel decisions.

#### 5.7.2. Module Complementarity

Figure 16 further illustrates the marginal gains of APF and HPI modules as functions of SNR. The marginal gain of the APF module increases monotonically with SNR (0.15 → 1.4), indicating that the adaptive fusion mechanism plays a greater role when channel quality is better. Conversely, the marginal gain of the HPI module remains stable in the low SNR range (approximately 0.2–0.35) but drops sharply after SNR exceeds 4.0 dB. The two modules exhibit significant complementarity, with the SNR range of 2.5–4.0 dB corresponding to maximized synergistic gains—a finding that provides quantitative guidance for operating point selection in practical systems.

#### 5.7.3. Future Directions

The above analysis reveals limitations in the current HPI design while indicating paths for improvement. A natural approach is to introduce adaptive mechanisms similar to APF into HPI, enabling the injection strength to respond to channel conditions. Specifically, one may consider introducing a learnable scaling factor $\alpha^{\left(l\right)}$ in each layer’s prior encoder, modulated by channel features: $h_{pr}^{\left(l\right)}=\alpha^{\left(l\right)}(f_{ch})\cdot ReLU(W_{pr}^{\left(l\right)}\cdot L_{src}+b_{pr}^{\left(l\right)})$ where $\alpha^{\left(l\right)}:R^{d}\to (0,1)$ is a lightweight gating network. This design maintains sufficient injection at low SNR while automatically attenuating at high SNR, thereby achieving optimal prior utilization across the entire SNR range.

This finding carries broader methodological implications: in neural network decoder design, the manner of introducing auxiliary information requires careful consideration of its dynamic relationship with primary information sources. Information fusion strategies should possess the capability to adaptively adjust based on the reliability of primary information.

## 6. Discussion

This work demonstrates that learnable mechanisms can effectively bridge the gap between the information-theoretic potential of non-equiprobable sources and practical decoding performance. Our findings extend prior research in neural network-enhanced decoding by addressing source statistic utilization—a dimension largely overlooked by existing Neural BP and GNN-based decoders.

The ablation study yields an important design insight: auxiliary information pathways benefit from SNR-aware adaptation. The contrasting behaviors of APF and HPI illustrate this principle. APF’s input-dependent gating inherently responds to channel quality, maintaining effectiveness across all SNR regimes. In contrast, HPI’s fixed-strength injection excels in low-SNR scenarios where prior information is most valuable. This complementarity, particularly evident in moderate SNR ranges, suggests that combining adaptive mechanisms at multiple stages offers significant potential for future decoder architectures.

From a deployment perspective, the computational speedup over conventional iterative decoders stems from the inherent parallelism of GNN operations, making the proposed architecture well-suited for GPU-accelerated implementations. The interpretable gate values further provide system designers with intuitive insights into decoder behavior, potentially enabling adaptive resource allocation in practical systems.

Several promising directions merit future investigation: an extension to fading channels and higher-order modulation schemes, the incorporation of the adaptive injection strength into HPI, and integration with emerging paradigms such as joint source-channel coding and semantic communications.

## 7. Conclusions

This paper presented the PE-HGNN, a Prior-Enhanced Heterogeneous Graph Neural Network decoder that addresses the fundamental challenge of exploiting non-equiprobable source statistics in LDPC decoding. Unlike conventional approaches that either ignore prior information or employ fixed fusion strategies, the proposed architecture systematically solves prior utilization problems through three synergistic innovations: Adaptive Prior Fusion for dynamic channel-prior balancing based on relative reliability, Heterogeneous Bipartite Message Passing for structure-aware feature propagation that explicitly distinguishes variable nodes from check nodes, and Hierarchical Prior Injection for sustained prior utilization across network depth. Experimental results on IEEE 802.11n LDPC codes demonstrate that the PE-HGNN consistently outperforms conventional prior incorporation methods. The learned gate values exhibit SNR-adaptive behavior consistent with Bayesian optimality principles, providing valuable interpretability for practical system design.

## Figures and Tables

**Table 1:** Comparison between ENGNN and PE-HGNN.

Aspect	ENGNN	PE-HGNN (Ours)
Application	Radio resource management	LDPC decoding with prior info
Edge Variables	Yes—beamformers/power as edge outputs	No—scalar weights for message scaling
Edge-Update	Full edge-update with neighbor aggregation	Learnable scalar weights, shared across layers
Key Innovation	Edge-update mechanism with PE property	Adaptive Prior Fusion + hierarchical injection
Multi-Source Fusion	Not applicable	Core contribution—gated channel/prior fusion

**Table 2:** Comparison of existing information fusion strategies for prior-aided channel decoding.

Method	Fusion Strategy	Adaptivity	Limitation
Direct Addition	$L_{0}=L_{ch}+L_{prior}$	None	Assumes prior always reliable
Fixed Weighting	$L_{0}=\alpha L_{ch}+\beta L_{prior}$	None	Weights predetermined
Soft Combination	Reliability-based weighting	Limited	Reliability hard to estimate
Proposed	Learnable gated fusion	Fully adaptive	—

**Table 3:** Training configuration table.

Parameter	Setting
LLR clipping range	[−20, 20]
Optimizer	Adam
Initial learning rate	0.001
Batch size	128
Epoch	300

**Table 4:** IEEE 802.11n LDPC code parameters.

Parameter	Symbol	Value
Code length	n	576
Information bits	k	432
Parity bits	m	144
Code rate	R	3/4

**Table 5:** Baseline decoder configurations for performance comparison.

Decoder	Configuration	Purpose
BP-50	Standard BP with 50 iterations	Performance upper bound of traditional decoding
BP-5	BP with 5 iterations	Fair comparison under equivalent complexity
BP+Prior	BP initialized with combined LLRs	Classical prior incorporation baseline
Homo-GNN	Homogeneous GNN with uniform node updates	Validate necessity of heterogeneous modeling

**Table 6:** FLOPs and parameter breakdown of PE-HGNN.

Component	FLOPs	%
APF Module	9.92 M	10.4%
HBMP × 5 layers	85.06 M	88.8%
HPI × 5 layers	0.74 M	0.8%
Output layer	0.07 M	0.1%
Total	95.79 M	100%

## Data Availability

The data that support the findings of this study are available from the corresponding author upon reasonable request.
